# When context modulates the influence of action observation on language processing

**DOI:** 10.1371/journal.pone.0201966

**Published:** 2018-08-08

**Authors:** Sophie-Anne Beauprez, Lucette Toussaint, Christel Bidet-Ildei

**Affiliations:** 1 Département de Psychologie, Université de Poitiers, Université de Tours, Centre National de la Recherche Scientifique, Centre de Recherches sur la Cognition et l’Apprentissage (UMR 7295), Poitiers, France; 2 Département des Sciences du sport, Université de Poitiers, Université de Tours, Centre National de la Recherche Scientifique, Centre de Recherches sur la Cognition et l’Apprentissage (UMR 7295), Poitiers, France; Universita degli Studi di Udine, ITALY

## Abstract

Numerous studies in the field of embodied cognition have shown a crosstalk between language and sensorimotor processes. In particular, it has been demonstrated that perceiving an action influences subsequent language processing. However, when studying the effect of action observation on language processing it has not been considered whether the context of action presentation could modulate this influence. To test this assumption, the participants in our study observed a prime, specifically a cartoon picture of a person performing an action in either a usual or an unusual context, and then had to perform a semantic decision task involving action verbs that could be congruent or incongruent with the action in the prime. Data analyses showed a significant difference on response times for congruent action verbs compared with incongruent action verbs in the usual context, whereas no difference was observed in the unusual context. This finding indicates that the influence of action observation on language appears only with usual actions, suggesting that the context of action presentation is crucial to enable the influence of action observation on action verbs processing.

## Introduction

The theory of embodied cognition suggests that human cognition is deeply rooted in the sensorimotor system [1]. According to this perspective, higher cognitive abilities depend on the activation of sensory and motor representations [2]. Specifically, with regard to language processing, embodied cognition theories suggest that semantic knowledge relies upon sensorimotor representations and that retrieving this semantic knowledge requires neural systems that are involved in the actual execution of the action [3, 4]. Therefore, action word processing could depend on the activation of motor processes that are involved in the actual performance of these actions [5].

Several studies confirmed this view by demonstrating that the sensorimotor system plays a role in the comprehension and production of language describing actions [6] and that executing an action could influence language processes [7, 8]. Moreover, the results of numerous brain imaging studies also support the existence of this action-language relationship [4], demonstrating that motor regions of the brain are active during the comprehension of action words [9] or of sentences involving actions [10]. Interestingly, the activations of motor system related to action verbs processing are somatotopic. Actually, Tettamanti and collaborators [10] observed brain activation in ventral regions in association with sentences describing mouth actions, whereas brain activations in association with sentences describing hand actions were in medio-dorsal regions and brain activations in association with sentences describing leg actions were in dorsal regions (see also [3, 9]). In the whole, these studies suggest that understanding action language implies an internal simulation of the sensorimotor representation involved in the execution of the action [4].

Interestingly, the majority of studies on the action-language relationship have focused on action execution and only few studies considered how action perception influences language processes. Yet it seems important to consider action perception paradigm, since it offers the possibility to assess specifically the central processes implied in the action-language relationship, and so, allows to differentiate between the influence of central processes (involved in the execution paradigms) and the influence of peripheral processes.

Studies have shown that action perception is related to language processes. Indeed, using fMRI [5, 11], similar brain activations were found when participants observed actions or read phrases involving actions and these activations were dependent of the effector of the action (hand, foot or mouth actions). Behavioral experiment also demonstrated that perceiving an action could modify subsequent language processes. In a study, Liepelt and collaborators [12] observed that participants were faster to say “open” according to a color of a square when this square was presented with a picture of an open hand and faster to say “close” with a picture of a closed hand. In the same vein, we recently demonstrated that observing a point-light human action facilitated the subsequent processing of congruent action verbs [13] suggesting that action observation and action language processing are based on the activation of common sensorimotor representations (see also [14, 15]).

However, these behavioral studies used isolated action (picture of a hand on a black background in the first case and point-light display in the second case) which does not reflect ecological situations where actions are always included in a context. This led us to question specifically the role of the context on the link between action observation and action verbs processing. We know that actions are considered “usual” only when they are presented in a typical and expected context, whereas actions that do not fit a given context are labelled “unusual” [16]. It has been demonstrated that the perception of unusual actions does not activate the same cerebral areas as the perception of usual actions [17]. In particular, the mirror network, which sustains action observation and action execution [18], appears to be insufficient to understand unusual actions. As some authors postulate that the mirror network plays a key role in the relationship between the sensorimotor system and language processing [5, 19], it can be assumed that the context of action presentation (usual versus unusual) could impact the action-language relationship.

The aim of the present study was to assess this assumption in order to deepen the understanding of the specific mechanism linking action perception and language processing. To do so, we examined whether the context of an observed action can affect the subsequent processing of an action verb. We used pictures depicting actions that were either usual or unusual. The unusual pictures were made so that the action would still be recognizable but would not fit the situation. For example, in a context of a person next to a plant, the action “watering” could be afforded whereas with a person next to a computer the action “watering” would not be afforded by the context. If the context of an action is a determinant characteristic, we hypothesize that the influence of action observation on language should decrease when the observed action is presented in an unusual context (e.g., watering a computer) compared with a usual context (e.g., watering a plant).

## Method

### Participants

Twenty-four French-speaking 19- to 22-year-old (M = 20, SD = 1.04) university students (17 male, 23 right-handed) participated in this experiment. The sample size was calculated using G*Power 3.0.10 [20]. It was based on a repeated-measures ANOVA design from the results obtained in a pilot study (Cohen’s d value = 0.84, correlations between repeated measures = 0.5). Statistical significance was set at p < .05 and power at .90. Participants were recruited in exchange for course credit. All participants had normal or corrected-to-normal vision and had no history of motor, perceptual or neurological disorders. The study is conformed to the Declaration of Helsinki. The ethical committee of the Research Centre on Cognition and Learning approved this study of human participants. All participants provided informed written consent before their participation. Before their participation, they were also unaware of the purpose of the study.

### Apparatus

The participants sat in a chair in front of a table in a dimly lit room. A computer (spatial resolution of 1280 pixels * 800 pixels and temporal resolution of 60 Hz) was on the table. A response box was placed on the table between the participants and the computer screen so the participants could easily provide their responses by pressing the button associated with a “yes” or “no” answer.

### Stimuli

**Fig 1:**
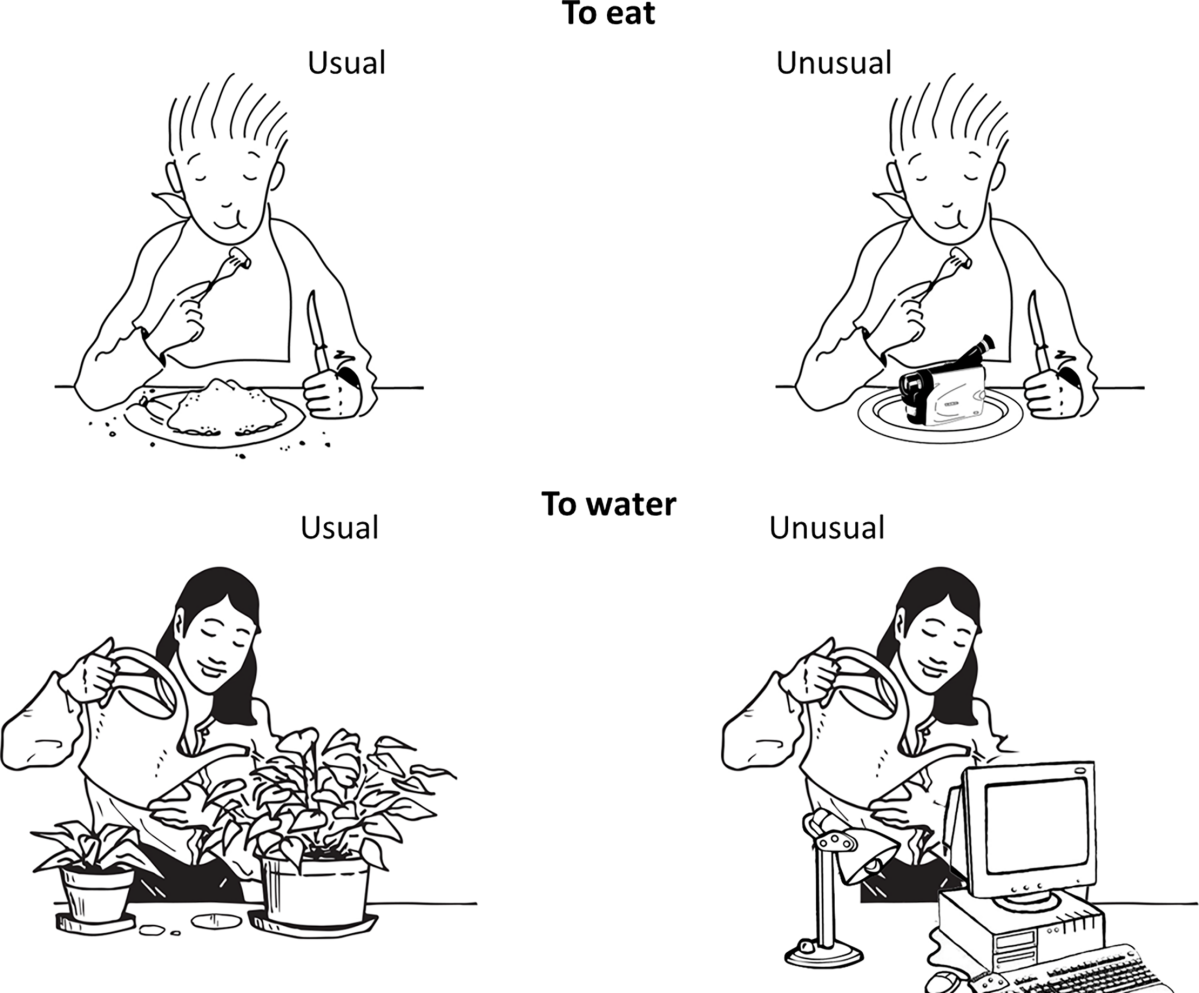
Examples of usual and unusual pictures used in the experiment. Usual actions have been selected in the French database Clic images 2.0 which proposes royalty-free illustrations (see Clic-Image2-0 –Réseau Canopé http://www.cndp.fr/crdp-dijon/clic-images/). Unusual actions have been built from the same model of character but were modified.

The prime was a black and white cartoon picture of a character performing an action with a size of 890 x 622 pixel. Ten different actions were used (to drink, to eat, to fish, to jump, to shoot, to ski, to swim, to walk, to water, to write). Each action was presented both in a usual context and in an unusual context, leading to a set of 20 pictures (see Fig 1 for examples and Table 1 for a description of each action in both contexts). The choice of these pictures was determined by a pilot study. An online questionnaire containing a set of pictures was completed by 34 people. They had to spontaneously name the action performed by the character by giving one or several verbs corresponding to it. We considered that the action was recognized when the corresponding verb was proposed by the majority of the participants. They also had to judge the plausibility of the action on a 5-point scale ranging from “very probable” to “very improbable”. The ten actions we kept had a mean plausibility score that was high for the usual context (4.8) and low for the unusual context (1.5). Moreover, these actions were recognized by more than 93% of the participants in both the usual and unusual context. Moreover, we carried a pilot study on 15 subjects. The aim of this study was to assess the time required to recognize the actions depicted by these pictures. The results showed that in mean, 1224 ms (SD = 514 ms) were necessary to recognize the usual pictures and 1241 ms (SD = 448 ms) for the unusual pictures. A Student’s t test revealed that this time of recognition was not significantly different between the two context (*p* = 0.8). The results of this pilot study and of the questionnaire allowed us to consider our prime pictures as equivalent in term of recognition.

**Table 1:** Description of the prime pictures and list of verbs.

Action of the prime	Human Action verb		Animal Action Verb
	Congruent Verb	Incongruent Verb	
Arroser (to water) Usual: a woman watering a plant Unusual: a woman watering a computer	To water	To write	To gallop To lay (an egg)
Boire (to drink) Usual: a man drinking water from a bottle Unusual: a man drinking poison from a bottle	To drink	To water	To hatch To meow
Ecrire (to write) Usual: a woman writing on paper Unusual: a woman writing on a pig’s back	To write	To eat	To forage To peck
Manger (to eat) Usual: a man eating food on a plate Unusual: a man eating a camera on a plate	To eat	To swim	To gallop To growl
Nager (to swim) Usual: a woman swimming in water Unusual: a woman swimming in leaves	To swim	To walk	To bark To lay (an egg)
Pêcher (to fish) Usual: a man fishing in a lake Unusual: a man fishing in a street	To fish	To shoot	To forage To roar
Promener (to walk) Usual: a man walking a dog Unusual: a man walking a snail	To walk	To ski	To bleat To peck
Sauter (to jump) Usual: a woman jumping over a trash Unusual: a woman jumping over a car	To jump	To fish	To growl To meow
Shooter (to shoot) Usual: a man shooting a ball Unusual: a man shooting a rock	To shoot	To drink	To bark To hatch
Skier (to ski) Usual: a man skiing on mountains Unusual: a man skiing at the beach	To ski	To jump	To bleat To roar

Regarding the stimuli, 20 verbs were used. Half of them were typical “human action verbs” (e.g., to drink, to fish) while the other half were “animal action verbs” (e.g., to meow, to roar). All verbs were presented in French in the infinitive form. The verb appeared in the center of the screen with an 18 point size (see Table 1 for the list of verbs).

The prime was presented before the action verb. In the case of “human action verbs” (half of the trials), this prime could be congruent (for example, seeing a picture depicting the action of drinking before reading the word “drink”) or incongruent (for example, seeing a picture depicting the action of skiing before reading the word “drink”) with the action verb presented as a stimulus. In the other half of the trials, the prime was presented before an animal action verb. These trials with animal action verbs were not analyzed. They were included only to develop a task for participants. The presentation order of the trials was randomized across participants.

### Procedure

**Fig 2:**
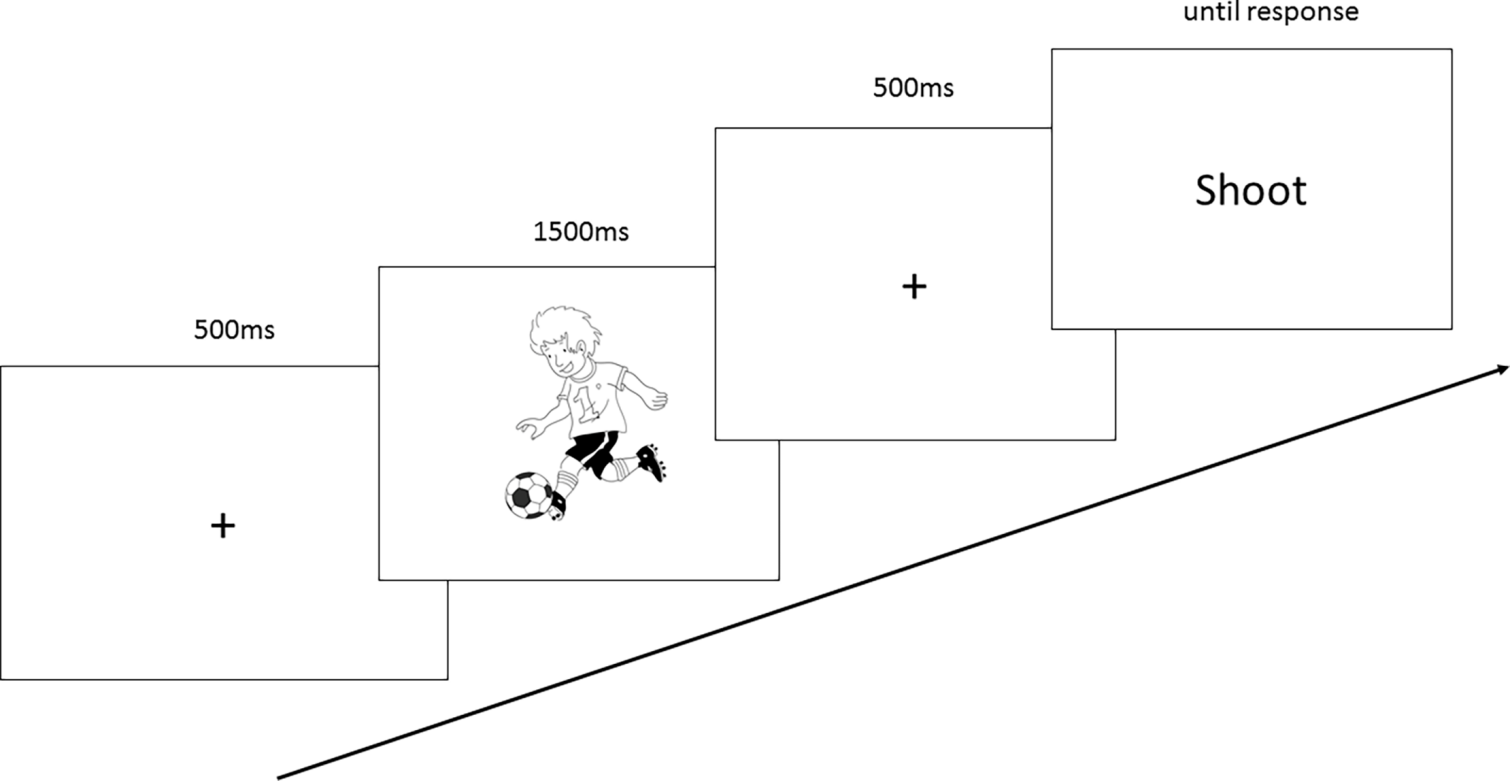
Procedure of the experiment task. The fixation cross, the prime picture as well as the verb stimulus were centered on the screen. The arrow represents the sequence of one trial. The image was one of the ten images extracted from Clic-image 2.0 selected in the French database Clic images 2.0 which proposes royalty-free illustrations (see Clic-Image2-0 –Réseau Canopé http://www.cndp.fr/crdp-dijon/clic-images/).

For each participant, the experimental session included 160 trials (10 pictures x 2 contexts (usual, unusual) x 4 verbs (human congruent/human incongruent/animal 1/animal 2) x 2 presentations. Each trial involved the following procedure (see Fig 2): a fixation cross appeared (500 ms), then, a picture (1500 ms) was presented. Finally, following another fixation cross (500 ms), the stimulus (a word) appeared. The stimulus remained on the screen until the participant entered a response. The participant’s task was to judge, as quickly and as accurately as possible, whether the word depicted a human action. Participants consistently entered a “yes” response with their dominant hand, whereas they entered a “no” answer with their other hand. The experiment lasted half an hour, with a break at the halfway point of the experiment.

After the participants completed the experimental task, two short questionnaires were administered to them. The aim of these questionnaires was to check whether each action in the primes had been recognized by the participants and assess whether the actions in the pictures actually appeared to be relatively probable according to their context.

The first questionnaire contained the twenty pictures in the task, and the participant had to say what action was depicted in each picture. To do so, the participant was asked to give one (or several) verb corresponding, according to him, to the action performed by the character. The picture obtained the score 1 when the answer provided by the participant corresponded to the action, namely when the participant gave the exact verb of the action or a semantically close verb (e.g., “to hop” instead of “to jump”). The picture obtained the score 0 when the answer provided by the participant differed semantically from the one expected (e.g., “to play” instead of “to shoot”). Then a percentage of recognition was calculated.

In the second questionnaire, the participant had to assess the plausibility of each picture in the task on a 5-point scale ranging from “very probable” to “very improbable”.

### Data analysis

Participants’ response time and accuracy for trials with human verbs were recorded. For response time, only correct answers were analyzed (98% of the data). Moreover, response time outliers (± 2.5 standard deviations) were excluded from the analysis (less than 2% of the data). Since our data follow a normal distribution (Shapiro-Wilk W test, *p* > 0.20), we used the lmer function of the lme4 package [21] in R environment (R version 3.3.0) to perform linear mixed-effects models. Participants and words items were specified as random-effects factors. Two fixed-effects factors were included: the picture context (usual action x unusual action) and the type of verb (congruent action x incongruent action), as well as their interaction. The *p* values were obtained reporting F values (Type III ANOVA) with error degree of freedom calculation based on Satterhwaite’s approximation. The responses of both questionnaires were assessed with paired Student’s t tests. The significance level was set at *p* < 0.05.

## Results

### Experimental task

**Fig 3:**
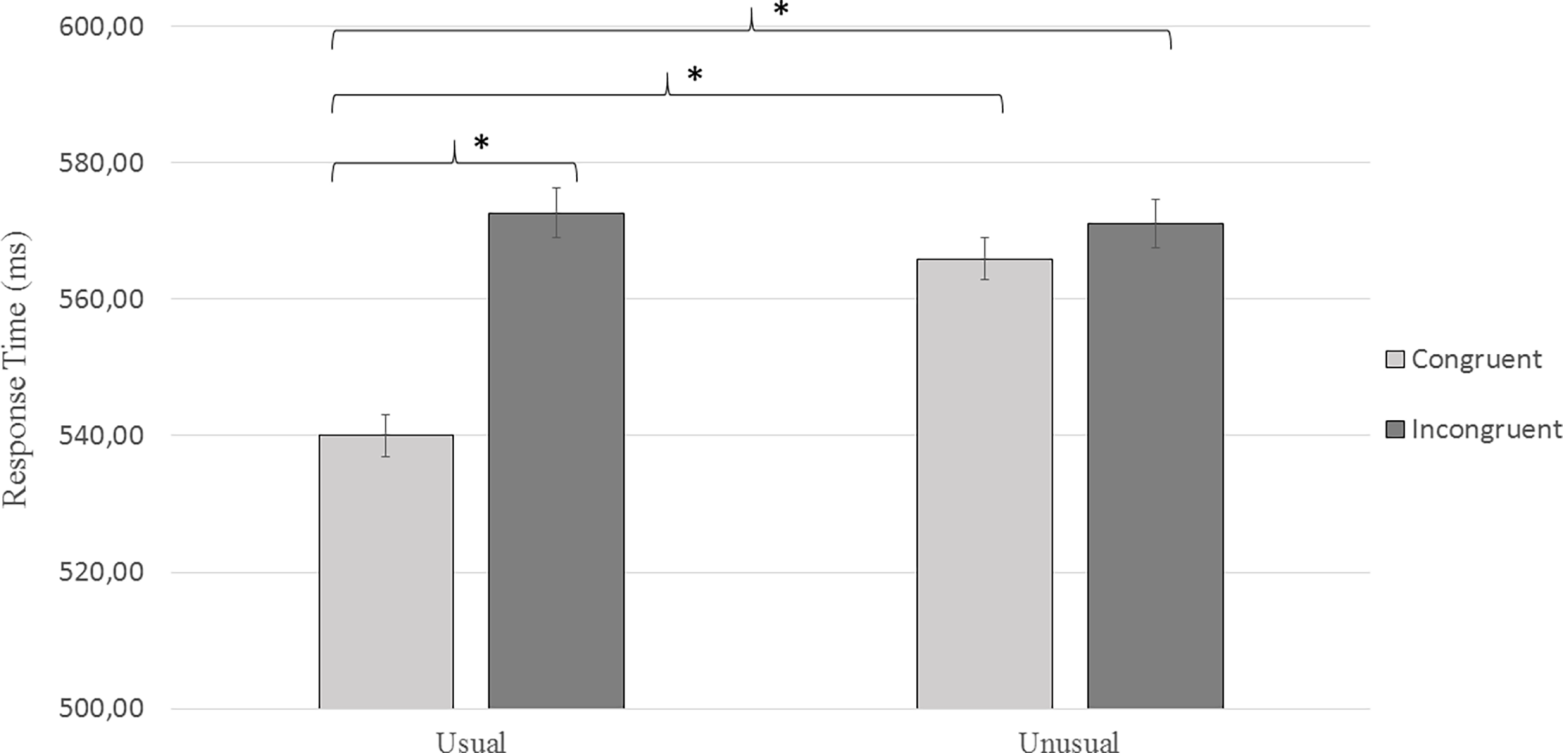
Mean response time according to the type of verbs (congruent action, incongruent action) and context (usual and unusual). The error bars indicate the 95% confidence interval. * significant difference with *p* < 0.05.

Given the high accuracy rate for each type of stimulus (> 97%, SD = 2.29), data analyses involved only the response time. The analyses showed that response time (see Fig 3) varied according to the type of verbs (F(1,1781) = 19.05; *p* < 0.01) and context (F(1,1781) = 7.72; *p* < 0.01). Moreover, a significant interaction between the type of verbs and context was found (F(1,1781) = 7.45; *p* < 0.01). The response time for usual congruent action verbs (M = 540.03 ms, SD = 71.17 ms) was significantly shorter than that of the usual incongruent action verbs (M = 572.64 ms, SD = 85.7 ms, *p* < 0.01), of the unusual congruent action verbs (M = 565.90 ms, SD = 79.45 ms, *p* < 0.01) and of the unusual incongruent action verbs (M = 571.09 ms, SD = 78.26 ms, *p* < 0.01). However, response time for usual incongruent action verbs, unusual congruent action verbs and usual incongruent action verbs were not significantly different (*p* > 0.5 each).

### Recognition questionnaire

Each action that was depicted in the pictures was recognized, regardless of the context of the presentation (T_23_ = 0.44; *p* = 0.66). In average, both the usual pictures and the unusual pictures were associated with a high recognition rate (98% for both, SD = 3.14 for the usual pictures and SD = 2.5 for the unusual pictures).

### Plausibility judgment questionnaire

Participants’ judgment of plausibility differed, according to the context of the actions (T_23_ = 46.35; *p* < 0.001). Usual action verbs were judged as being more probable (M = 4.7, SD = 0.2) than unusual action verbs (M = 1.4, SD = 0.4).

## Discussion

The aim of the present study was to assess whether the context of an observed action can affect the relationship between language and the sensorimotor system. We presented to the participants cartoon pictures depicting an unusual or a usual action before they completed a language decision semantic task. Our results showed that observing a picture of an action influenced the processing of the verb related to this action but only when the context of this action was usual. That is, no effect was observed in association with the pictures of unusual actions. In a previous study, van Elk, van Schie and Bekkering [22] showed that only performing usual actions (e.g., bringing a cup to the mouth) provided the appropriate context for processing congruent actions words (less negative N400-amplitude) compared with performing unusual actions (e.g., bringing a cup to the ear). Our results confirm the importance of context in action-language relationship and extend previous findings even when the action is not executed but only observed.

A possible interpretation of our results could simply be a decrease in action recognition when the context is unusual. That is, if the action is recognized to a lesser degree, it could be reasonable to think that the strength of the action-language relationship should decrease. However, the results obtained with regard to recognition do not support this view because we did not find any difference between action recognition in usual and unusual contexts. In the same manner, we can consider that these results are due to difficulties of the participants to process unusual pictures in the allotted time. This possibility is also unlikely because the results of our pilot study has demonstrated that usual and unusual pictures are semantically processing in the same time window (1250 ms in average). It is more probable that the absence of effect in association with a congruent unusual action is related to a decrease in sensorimotor activation. Since sensorimotor representations are involved when an observed action matches the perceiver’s motor repertoire [23], we assume that unusual actions are unable to activate sensorimotor activation or at least that such an activation is weaker than that which is usually produced by an observation of usual actions [17]. Several authors have suggested that the action-language relationship is sustained by motor resonance [14, 24, 25], and it is known that motor resonance is related to the sensorimotor experience (e.g., [23]). Consequently, in the present study, we can speculate that the unusual action could not be related to the perceiver’s motor repertoire and, consequently, that they activated motor resonance to a lesser extent. Moreover, studies suggest also that observation of object activates a motor simulation of the possible actions to perform with them [26] but that according to the context different affordances are activated. For example, participants were faster to answer to function and manipulation verbs after observing an object in the peripersonal space compared with this same object in the extrapersonal space [27]. In our experiment, we can assume that the unusual picture should not have activated the potential actions to perform with the object depicted (i.e., seeing a bottle of poison should not activate “to drink”). Thus, our results are in agreement with previous studies that suggested that motor resonance is modulated by the context of an observed action [28, 29]. For example, Amoruso and Urgesi [28], using TMS and recording motor evoked potentials, demonstrated that the corticospinal excitability decreased when an action was performed in an incongruent context (i.e., pouring when a glass of water is already full). Moreover, the performance in predicting action was impaired in comparison with when an action was performed in a congruent context (i.e., pouring an empty glass of water). Interestingly, Amoruso, Finisguerra and Urgesi [29] showed that the modulation of the motor resonance by the context could be related to an early facilitatory mechanism in the case of action perceived in a congruent context and to a later inhibitatory mechanism in the case of action perceived in an incongruent context. In the presented experiment, response times were shorter for the usual congruent action compared with all the other conditions. This could indicate that motor resonance was facilitated in this situation and that on the contrary the mechanism of motor resonance was inhibited for the unusual congruent action. However this would need to be assessed specifically in future studies.

Moreover, it might be proposed that the absence of effect in the unusual context could be related to the activation of a mentalizing system. Some authors have shown that the observation of a usual action is supported by the activation of the mirror neuron system [30] that plays a role in recognizing the action but also in coding the intention of this action [31]. However, for the unusual condition, research has shown that the activation of the mirror neuron system is not sufficient and that the intervention of another system, the mentalizing system, is necessary [17]. This system is known to be activated when mentalization and rationalization are required, specifically when inferences about goals, beliefs or moral issues must be made [32]. This idea is supported by cerebral studies on action perception in which the plausibility of the action was manipulated. For example, Liepelt, Von Cramon and Brass [33] asked participants to observe finger lifting movements that differed in plausibility while recording fMRI data. Their results suggested that the mirror neuron system was involved in understanding intentional action, whereas the mentalizing system was involved in inferring intention from non-stereotypical situations (see also [34]). Therefore, the mirror and mentalizing systems might be complementary systems that are involved in the understanding of action. On the one hand, the mirror network plays a role in usual and stereotypical situations in which no inferential processing is required. Therefore, for a usual context, the action is mapped onto the corresponding motor representations that are already present in the observer’s action schemes. On the other hand, the processing of unusual actions requires active inferencing from the participant. That is why unusual actions are mediated by regions that are related to rationalization mechanisms that are part of an inferential system, the mentalizing system [35]. The results of the present study could be interpreted in light of these data. We can hypothesize that the usual pictures presented to the participants could have activated the mirror neuron system, thereby activating the sensorimotor representation related to this action. In contrast, the unusual pictures presented to the participants could have activated the mentalizing neuron system and less activated the mirror neuron system; consequently, the sensorimotor representation would be reduced or not activated, which would have led to the absence of effect in the unusual context.

Finally, we could consider another explanation that would not involve motor simulation but instead that would only rely on perceptual simulation. Indeed, we can view language comprehension as a construction of a perceptual simulation to represent the meaning of the word or sentence [1] and it has been demonstrated that visual perception is able to influence language processing. For example, participants responded faster when they read a sentence suggesting implicitly a particular orientation of an object (“a pencil in a drawer” involving a horizontal orientation vs “a pencil in a cup” involving a vertical orientation) after observing an object matching the orientation of the sentence [36]. In our experiment, we can hypothesize that the perceptual simulation of the action would be affected by the context of depiction of the action. Indeed, the perceptual symbol system assume an analog relationship between a symbol and its referent and that changes in the referent will cause changes in the perceptual symbol [1]. The perceptual simulation could have been modified in the unusual context, consequently affecting the language task that required to perceptually simulate the action (to judge whether the verb is human-related or animal-related). To disentangle motor and perceptual interpretations, futures experiments should compare brain activity (EEG or fMRI) during the usual and unusual contexts.

It is worth noting that an effect of action observation on language processing was produced by the simple presentation of a static picture. This is not by itself surprising since previous studies already demonstrated that static pictures enabled the activation of the mirror neuron system [37]. This would be in agreement with our proposition of a motor resonance when perceiving a congruent action. However, what is more particular is that we demonstrated that people are influenced even if the action is performed by a cartoon character. Maybe, we could have expected different results to our study when showing pictures of a real person since it could be easier to resonate with real human being rather than with cartoon character.

## Conclusion

The present study shows that the context in which an action is perceived critically affect the link between action and language. This result brings us more insight on how action perception influences language processing, and this should be taken into account in the future.

In conclusion, this study demonstrates for the first time that the context of a perceived action influences action-verb processing. Future researches aims should be to determine whether motor simulation and/or perceptual simulation is responsible of the influence of action observation on language processes. Moreover, if motor simulation is involved, we will have to determine whether this modulation by the context could be explained by the intervention of two distinct networks involved in the understanding of unusual and usual actions (i.e., the mirror neuron system versus the mentalizing neuron system) or by a decrease in sensorimotor activation related to decreased motor resonance. Brain activation studies should be conducted to explore these questions more specifically.
